# *suz12* inactivation in *p53*- and *nf1*-deficient zebrafish accelerates the onset of malignant peripheral nerve sheath tumors and expands the spectrum of tumor types

**DOI:** 10.1242/dmm.042341

**Published:** 2020-08-27

**Authors:** Felix Oppel, Dong H. Ki, Mark W. Zimmerman, Kenneth N. Ross, Ting Tao, Hui Shi, Shuning He, Jon C. Aster, A. Thomas Look

**Affiliations:** 1 Department of Pediatric Oncology, Dana-Farber Cancer Institute, Harvard Medical School, Boston, MA 02115, USA; 2 Department of Pathology, Brigham and Women's Hospital, Harvard Medical School, Boston, MA 02115, USA

**Keywords:** MPNST, Leukemia, SUZ12, p53, NF1, RAS signaling

## Abstract

Polycomb repressive complex 2 (PRC2) is an epigenetic regulator of gene expression that possesses histone methyltransferase activity. PRC2 trimethylates lysine 27 of histone H3 proteins (H3K27me3) as a chromatin modification associated with repressed transcription of genes frequently involved in cell proliferation or self-renewal. Loss-of-function mutations in the PRC2 core subunit SUZ12 have been identified in a variety of tumors, including malignant peripheral nerve sheath tumors (MPNSTs). To determine the consequences of *SUZ12* loss in the pathogenesis of MPNST and other cancers, we used CRISPR-Cas9 to disrupt the open reading frame of each of two orthologous *suz12* genes in zebrafish: *suz12a* and *suz12b*. We generated these knockout alleles in the germline of our previously described *p53* (also known as *tp53*)- and *nf1-*deficient zebrafish model of MPNSTs. Loss of *suz12* significantly accelerated the onset and increased the penetrance of MPNSTs compared to that in control zebrafish. Moreover, in *suz12-*deficient zebrafish, we detected additional types of tumors besides MPNSTs, including leukemia with histological characteristics of lymphoid malignancies, soft tissue sarcoma and pancreatic adenocarcinoma, which were not detected in *p53/nf1-*deficient control fish, and are also contained in the human spectrum of *SUZ12*-deficient malignancies identified in the AACR Genie database. The *suz12*-knockout tumors displayed reduced or abolished H3K27me3 epigenetic marks and upregulation of gene sets reported to be targeted by PRC2. Thus, these zebrafish lines with inactivation of *suz12* in combination with loss of *p53/nf1* provide a model of human MPNSTs and multiple other tumor types, which will be useful for mechanistic studies of molecular pathogenesis and targeted therapy with small molecule inhibitors.

## Abstract (precis)

**Summary:** In *p53-* and *nf1*-deficient zebrafish, onset of MPNSTs, as well as diverse other tumors, is accelerated by loss of the *suz12* tumor suppressor, accompanied by global reduction in H3K27me3 marks and increased Ras-Mapk signaling.

## INTRODUCTION

Alterations in genes encoding epigenetic regulators of gene expression have become increasingly important in cancer biology. Polycomb group (PcG) proteins are key epigenetic regulators that interact with each other to form chromatin-modifying complexes. The major PcG complexes include polycomb repressive complex 1 (PRC1) and polycomb repressive complex 2 (PRC2) (Margueron and Reinberg, 2011), with PRC2 identified as an especially promising drug target for cancer therapy (Shi et al., 2017). PRC2 core components include the proteins SUZ12, EED, the histone methyltransferases EZH1 or EZH2, and the histone binding protein RbAp48 (also known as RBBP4) (Margueron and Reinberg, 2011). The PRC2 complex silences the expression of its target genes by catalyzing the methylation of lysine 27 in the tail of histone H3 family proteins (the H3K27me3 mark). SUZ12 is required for the structural integrity of the PRC2 complex and to facilitate chromatin binding (Chen et al., 2018). Additionally, the detailed mechanisms of PRC2-mediated gene silencing depend heavily on cellular context, as PRC2 targets different sets of genes in different cell types (Squazzo et al., 2006).

The function of PcG proteins was first described in *Drosophila* development, where PcG multisubunit complexes repress Hox genes, which are conserved regulators of cell identity within the anterior-posterior axis (Comet et al., 2016; Lewis, 1978; Jones and Gelbart, 1990; Simon et al., 1992; Struhl and Akam, 1985). It has also been demonstrated that the PcG complexes are important to maintain cell identity by keeping previously silenced genes silent, rather than newly initiating transcriptional repression (reviewed in Comet et al., 2016). In particular, during differentiation of embryonic stem cells, PRC2 is not required for the establishment of repressive marks at target genes, but rather is required for maintenance of gene silencing (Riising et al., 2014; Yuan et al., 2012; Hosogane et al., 2013).

Functional PRC2 is crucial for normal development and a complete loss of the core subunit Suz12 in mice is embryonically lethal (De Raedt et al., 2014). *SUZ12* is reported to be a tumor suppressor gene in malignant peripheral nerve sheath tumors (MPNSTs) and high-grade gliomas (HGGs) (De Raedt et al., 2014; Lee et al., 2014), while in epithelial ovarian cancer, breast cancer and other malignancies (Li et al., 2012; Kirmizis et al., 2003), it might function to promote oncogenesis. Thus, the role of SUZ12 in tumorigenesis has remained unclear, which is also true for the PRC2 subunit EZH2 (Hock, 2012). In neurofibromatosis type 1, loss of PRC2 activity reduces the levels of H3K27me3 and leads to elevated RAS-dependent transcription that facilitates transformation of benign plexiform neurofibroma precursor lesions into MPNSTs (De Raedt et al., 2014; Baude et al., 2014). *SUZ12* loss-of-function (LOF) has been shown to cooperate in tumorigenesis with combined loss of the RAS GTPase-activating protein (RASGAP) NF1 and the tumor suppressor p53 (also known as TP53) (De Raedt et al., 2014). Moreover, *SUZ12* loss can elevate expression of Hox genes such as *HOXC13* (Marcinkiewicz and Gudas, 2014), which is implicated in metastatic dissemination in melanoma (Cantile et al., 2012), another tumor in which *SUZ12* LOF often cooperates with *NF1* loss (De Raedt et al., 2014). In addition to solid tumors, *SUZ12* and *EZH2* were previously identified as tumor suppressor genes in leukemia (Ntziachristos et al., 2012). In T cell acute lymphoblastic leukemia (leukemia), loss of PRC2 core subunits was reported to occur by mutation or deletion in about 25% of all cases, and in a NOTCH1-induced genetic mouse model of leukemia, NOTCH1 antagonizes PRC2 function, leading to a loss of H3K27me3 (Ntziachristos et al., 2012).

In order to function properly, PRC2 requires the additional binding of the α-thalassemia/mental retardation syndrome X-linked protein (ATRX), which is crucial for directing PRC2 to maintain the state of genes already silenced by PRC1. In the absence of ATRX, the deposition of H3K27me3 is misplaced to ectopic sites in the intergenic space and at non-canonical sites in the target genes, which impairs the maintenance of silenced genes (Sarma et al., 2014). In a previous *atrx*-knockout model in zebrafish, we observed the re-expression of PRC2 target genes upon Atrx depletion, despite initial H3K27me3 deposition (Oppel et al., 2019).

In this study, we report the consequences of loss of *suz12* in a *p53/nf1*-deficient zebrafish tumor model that is suitable for drug testing (Ki et al., 2019; Shin et al., 2012; Ki et al., 2017). In our model, we have assessed *suz12* LOF-mediated carcinogenesis in a dose-dependent manner and translated our results based on current studies of human cancer genetics. We dissect the consequences of *suz12* depletion on oncogenic Ras-Mapk signaling and indicate MEK inhibition as an effective strategy in *p53*/*nf1*/*suz12-*deficient MPNSTs. Moreover, we present a model of *suz12* LOF-induced leukemia, soft tissue sarcoma and pancreatic adenocarcinoma, which will aid in preclinical studies of these diseases.

## RESULTS

### Knockout of *suz12a* and *suz12b* in the zebrafish germline

**Fig. 1.**
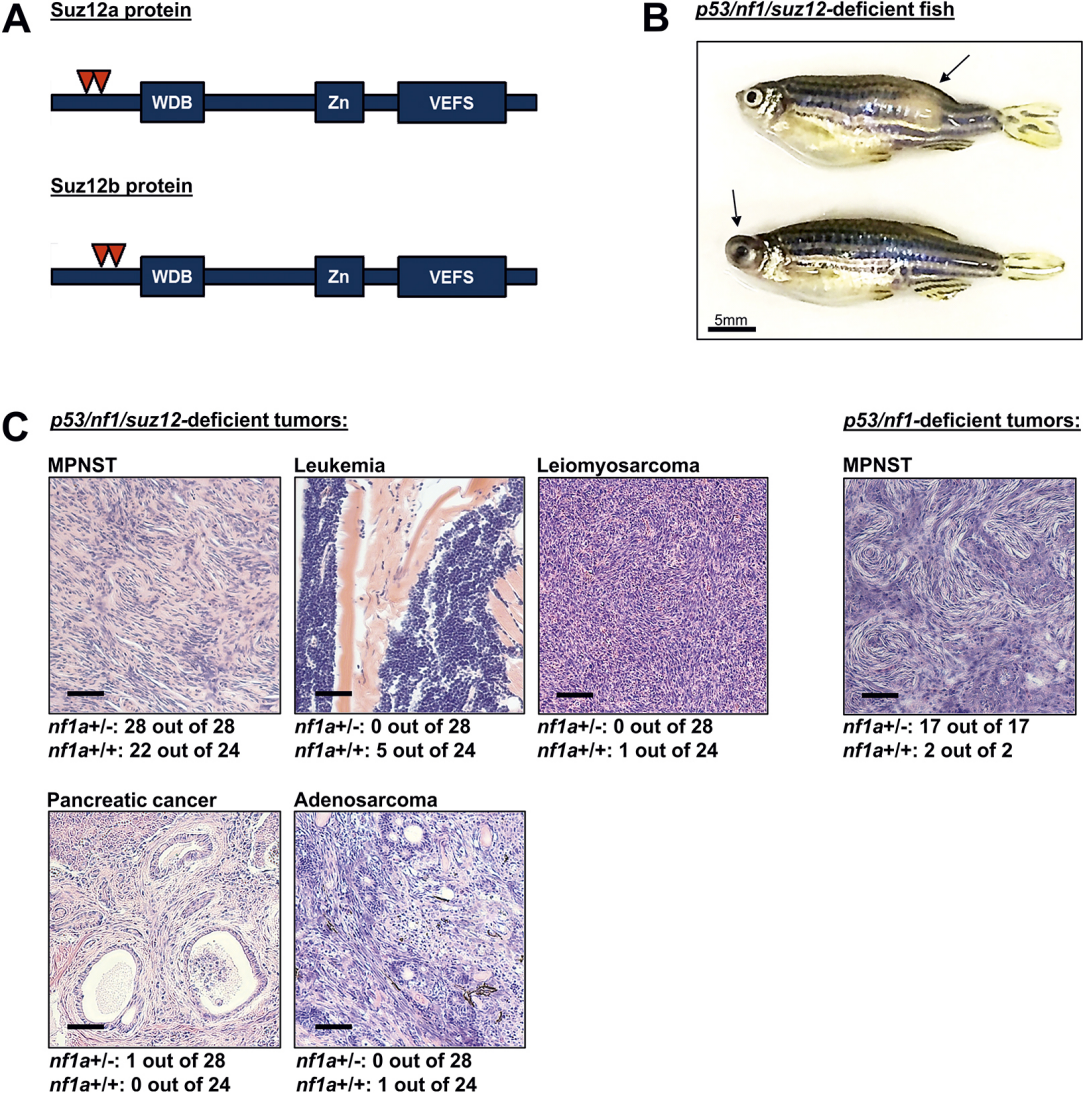
**Loss of *suz12* in *p53/nf1*-deficient fish diversifies carcinogenesis.** (A) CRISPR-Cas9-mediated targeting truncates Suz12a and Suz12b proteins before their functional domains, conferring a loss of function. VEFS, VRN2-EMF2-FIS2-SUZ12 domain; WDB, WD-40 binding domain; Zn, Zn-finger region. (B) *p53/nf1/suz12*-deficient fish are prone to tumors in various anatomical locations, e.g. the abdomen or head (arrows). (C) Histopathology analysis reveals a variety of cancer types in *p53/nf1/suz12*-deficient tumor-bearing fish, whereas *p53/nf1*-deficient control fish solely develop MPNSTs. Numbers underneath representative images indicate the frequency of the respective tumor type in *nf1a^+/−^* or *nf1^+/+^* tumor-bearing fish analyzed by histology. Scale bars: 100 µm.

To create knockout mutations in the *suz12* tumor suppressor gene using CRISPR-Cas9, we designed sgRNAs to target exon 1 directly after the start of the coding sequence (Fig. 1A). Because zebrafish harbor two *suz12* paralogs (*suz12a* and *suz12b*), sgRNAs targeting both genes, and *Cas9* mRNA, were injected into one-cell embryos derived from a previously established *p53/nf1*-deficient line expressing a GFP marker gene under the control of the endogenous *sox10* promoter, Tg(*sox10*:GFP) (Shin et al., 2012), or from wild-type (AB strain) zebrafish. In the *p53/nf1*-deficient background, we employed two sets of sgRNAs targeting exon 1 of *suz12a* and *suz12b* sharing no sequence identity. In the AB background, sgRNA pairs were used to generate a *suz12* knockout independently of *p53/nf1* deficiency. This procedure efficiently resulted in germline mutations, which were passed from primary injected F0 zebrafish into the F1 generation (Table S1). In 13 tested F1 fish, both sgRNAs targeting *suz12b* exclusively induced small deletions (2 bp-8 bp) resulting in a frameshift mutation, whereas at the *suz12a* locus we observed both small deletions (1 bp-9 bp) and insertions (1 bp-18 bp). In the *p53/nf1*-deficient background, total loss of *suz12a/b* was lethal in developing embryos, beginning between 8 and 15 days postfertilization (dpf), so at least one allele of either *suz12a* or *suz12b* was retained in adult fertile *p53/nf1/suz12*-mutant fish.

### Loss of *suz12* diversifies carcinogenesis

Our previously established model based on combinatorial loss of *p53* and *nf1* is prone to gliomas at low penetrance and MPNSTs at high penetrance (Shin et al., 2012). As with *suz12*, the *nf1* gene is duplicated in zebrafish, resulting in two *nf1* paralogs termed *nf1a* and *nf1b*. Because a total loss of *nf1* is lethal in developing fish, one allele of *nf1a* is preserved, which after inbreeding leads to a mixed population of *p53^m/m^, nf1b^−/−^, nf1a^+/^^−^* and *p53^m/m^, nf1b^−/−^, nf1a^+/+^* progeny. Zebrafish with an *nf1a^+/−^* genotype have a much faster tumor onset than *nf1a^+/+^* siblings. To assess the biological impact of introducing a *suz12* LOF mutation, we monitored tumor onset and penetrance in developing offspring. Zebrafish harboring *suz12* mutations of both genotypes developed tumors in abdomen, head, tail and anal sites (Fig. 1B) that were visually indistinguishable from the MPNSTs arising in the original *p53/nf1*-deficient line (Shin et al., 2012).

Histopathologic analysis of paraffin-embedded tumor tissue revealed that the *suz12* disruption diversified the spectrum of tumor types considerably (Fig. 1C, Table 1). In *p53^m/m^, nf1b^−/−^, nf1a^+/^^−^* and *p53^m/m^, nf1b^−/−^* and *nf1a^+/+^* control fish, only MPNSTs were detected. In the *p53/nf1/suz12-*mutant line, we observed MPNSTs as well as leukemias with histological characteristics of lymphoid malignancies, pancreatic adenocarcinoma, a soft tissue sarcoma in the head region, and one case of a mixed mesenchymal/epithelioid tumor in the tail that was not further definable and therefore was referred to as adenosarcoma (Fig. 1C, Table S2). In the *p53^m/m^, nf1b^−/−^, nf1a^+/^^−^, suz12-*mutant cohort, all 28 tumor-bearing fish that were sectioned displayed MPNSTs, one of which also displayed the sole case of pancreatic adenocarcinoma (3.6%). In the *p53^m/m^, nf1b^−/−^, nf1a^+/+^, suz12-*mutant population, 22 of the 24 tumor-bearing fish (91.7%) had MPNSTs, five displayed leukemia (20.8%), and a single fish showed soft tissue sarcoma or adenosarcoma (4.2%) (Table S2). Interestingly, six out of 52 analyzed fish simultaneously carried multiple distinct tumor lesions (11.5%). In the wild-type background, we did not detect MPNST or any of the additional tumors present in *suz12*-deficient zebrafish after at least 1 year of monitoring. Thus, the loss of *suz12* alone was insufficient to drive tumorigenesis in our model within the time frame of our analysis.

**Table 1.**
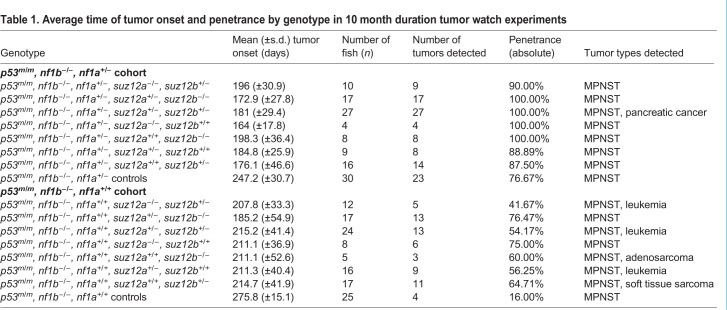
**Average time of tumor onset and penetrance by genotype in 10 month duration tumor watch experiments**

### Loss of *suz12* accelerates tumorigenesis

**Fig. 2.**
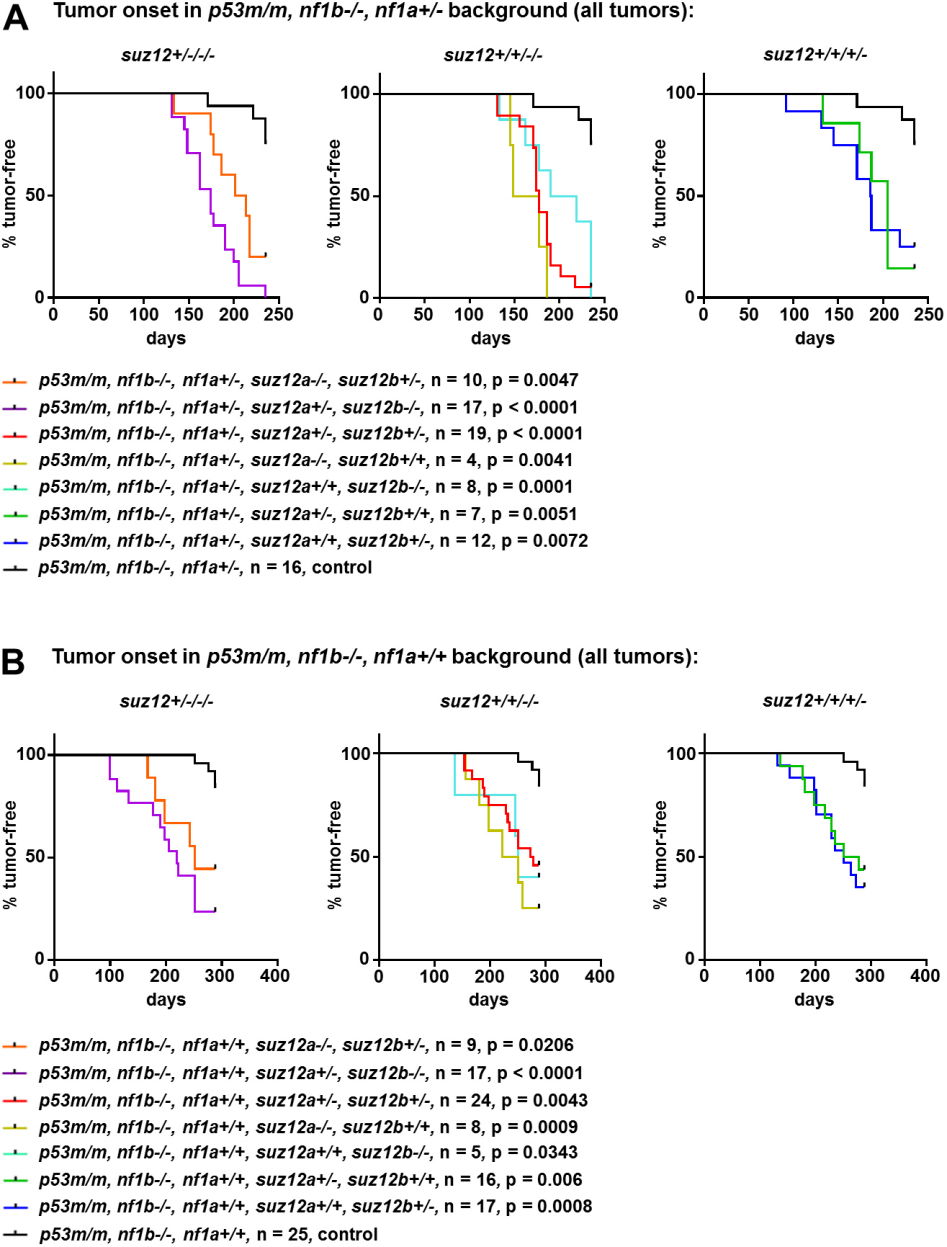
**Tumor watch of *suz12*-mutant fish in the *p53^m/m^, nf1b^−/−^, nf1a^+/^^−^* and** ***p53^m/m^, nf1b^−/−^, nf1a+/+* backgrounds.** Representative experiments are displayed to show tumor onset time for fish with *nf1a*^+/−^ genotype (A) or *nf1a^+/+^* genotype (B), plotted by *suz12* genotype; either three out of four (*suz12*^+/−/−/−^; left), two out of four (*suz12*^+/+/−/−^; middle), or one out of four (*suz12*^+/+/+/−^; right) *suz12* alleles are knocked out. The data from each knockout population was compared to the respective *suz12*-wild-type control population to calculate *P*-values using Student's *t*-tests.

Tumor onset was markedly accelerated overall in both the *p53^m/m^, nf1b^−/−^, nf1a^+/^^−^* (Fig. 2A) and the *p53^m/m^, nf1b^−/−^, nf1a^+/+^* backgrounds (Fig. 2B) upon the depletion of *suz12*. Acceleration of tumor onset in *suz12-*mutant zebrafish compared to wild-type controls were significant for all *suz12*-mutant populations, independent of whether one (*suz12*^+/+/+/−^), two (*suz12*^+/+/−/−^) or three (*suz12*^+/−/−/−^) alleles in any combination were disrupted. Besides the faster onset, the proportion of tumor-bearing fish (penetrance) was increased in *p53/nf1/suz12*-knockout fish compared to that in controls (Fig. 2, Table 1).

**Fig. 3.**
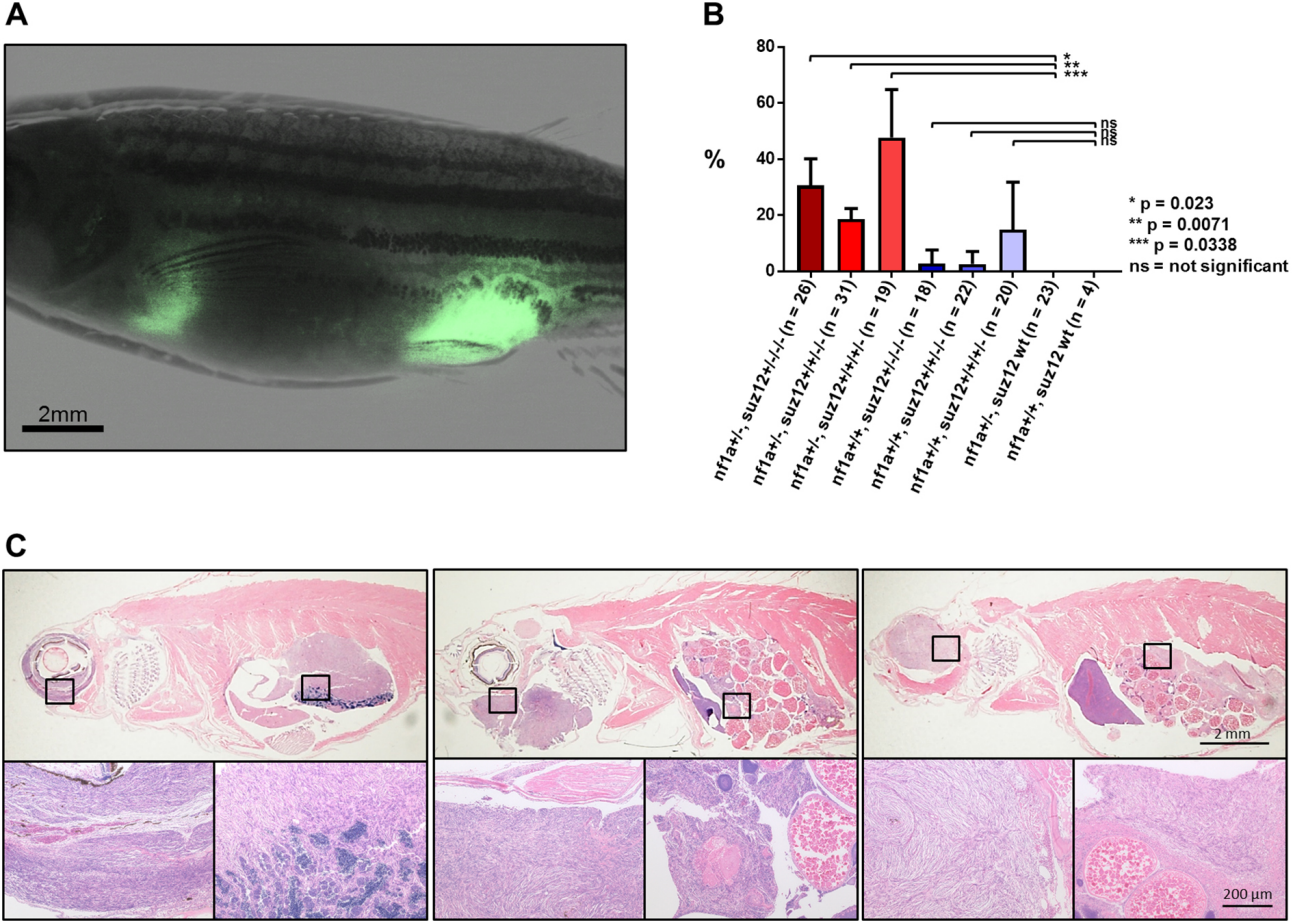
**Observation of multiple tumor foci in *p53/nf1/suz12*-deficient zebrafish lines.** (A) Fluorescence microscopy image of an adult *p53^+/−^, nf1b^−/−^, nf1a^+/^^−^, suz12a^+/^^−^, suz12b^+/^^−^* fish bearing two independent GFP-positive tumors in abdominal and anal locations. (B) Proportion of fish with multiple lesions in different *suz12*-mutant populations with additional *p53^+/−^, nf1b^−/−^, nf1a^+/^^−^* or *p53^+/−^, nf1b^−/−^, nf1a^+/+^* backgrounds. Data are mean±s.d. of populations with any combination of three out of four (*suz12*^+/−/−/−^), two out of four (*suz12*^+/+/−/−^), or one out of four (*suz12*^+/+/+/−^) *suz12* alleles knocked out. The total number of fish analyzed is indicated (*n*). *P*-values were calculated using Student's *t*-tests. (C) Representative HE-stained sections of *p53/nf1/suz12*-deficient fish with two separate tumor foci. Squares indicate areas shown in magnified images below.

A significant proportion of *p53/nf1/suz12*-mutant fish developed multiple tumor foci that were cleanly distinguishable by the expression of the *sox10*:GFP marker gene (Fig. 3A). In all *p53^m/m^, nf1b^−/−^, nf1a^+/−^, suz12*-mutant populations, multiple tumor foci were observed in 10-70% of the tumor-bearing fish. In *p53^m/m^, nf1b^−/−^, nf1a^+/+^, suz12*-mutant fish the incidence of multiple tumors was much lower, in the range of 0-35%. Again, the *suz12a^+/−^, suz12b^−/−^*; *suz12a^+/+^, suz12b^−/−^*; and *suz12a^+/+^, suz12b^+/−^* populations were not significantly different from each other. The tumor onset in the *suz12a^+/−^, suz12b^−/−^* and *suz12a^+/+^, suz12b^−/−^* populations of the *nf1a*^+/−^ cohort were both significantly different from the *suz12a^+/+^,* *suz12b^+/+^* fish (*P*<0.0005) (Fig. 3B). Because of the low incidence of multifocal tumors in the *nf1a^+/+^* cohort, loss of *suz12* did not significantly affect tumor onset. Importantly, although multifocal tumors were observed in all *suz*12-deficient populations, they never arose in *suz12*-wild-type fish in the *nf1b^−/−^,* *nf1a^+/−^* background. The *suz12*-deficient fish with multiple tumor foci either presented distinct malignancies (e.g. leukemia and MPNST; Table S2) or multiple MPNST foci in distinct anatomic locations, so that a clear separation of these foci could be confirmed by histology (Fig. 3C).

### The *suz12*-deficient tumor model in zebrafish is consistent with human tumor genetics

As emphasized above (Fig. 1C), the spectrum of tumorigenesis was diversified after disruption of *suz12* in the *p53/nf1*-deficient background. To determine whether the additional tumor types were consistent with those observed in human patients, we examined the *SUZ12* mutant sample cohort of the AACR Genie database (AACR Project GENIE Consortium, 2017). Mutations in *SUZ12* are annotated in 35 tumor types in the AACR Genie database (v4.0), which include MPNST, pancreatic cancer, leukemia and soft tissue sarcoma (Table S3). The most frequently recorded *SUZ12*-mutated or *SUZ12*-deleted cancer type category is ‘nerve sheath tumor’, including MPNSTs (5.56% and 4.21%, respectively). Pancreatic cancer, leukemia and soft tissue sarcoma were also found within the sample cohort of the database for combined *p53/SUZ12* mutations (AACR Project GENIE Consortium, 2017). Thus, the tumor types we identified in *suz12*-deficient fish are consistent with the malignancy spectrum in human patients that emerges from analysis of the AACR Genie database.

### Tumors in *suz12*-deficient zebrafish display decreased H3K27me3 and elevated Ras-Mapk signaling

**Fig. 4.**
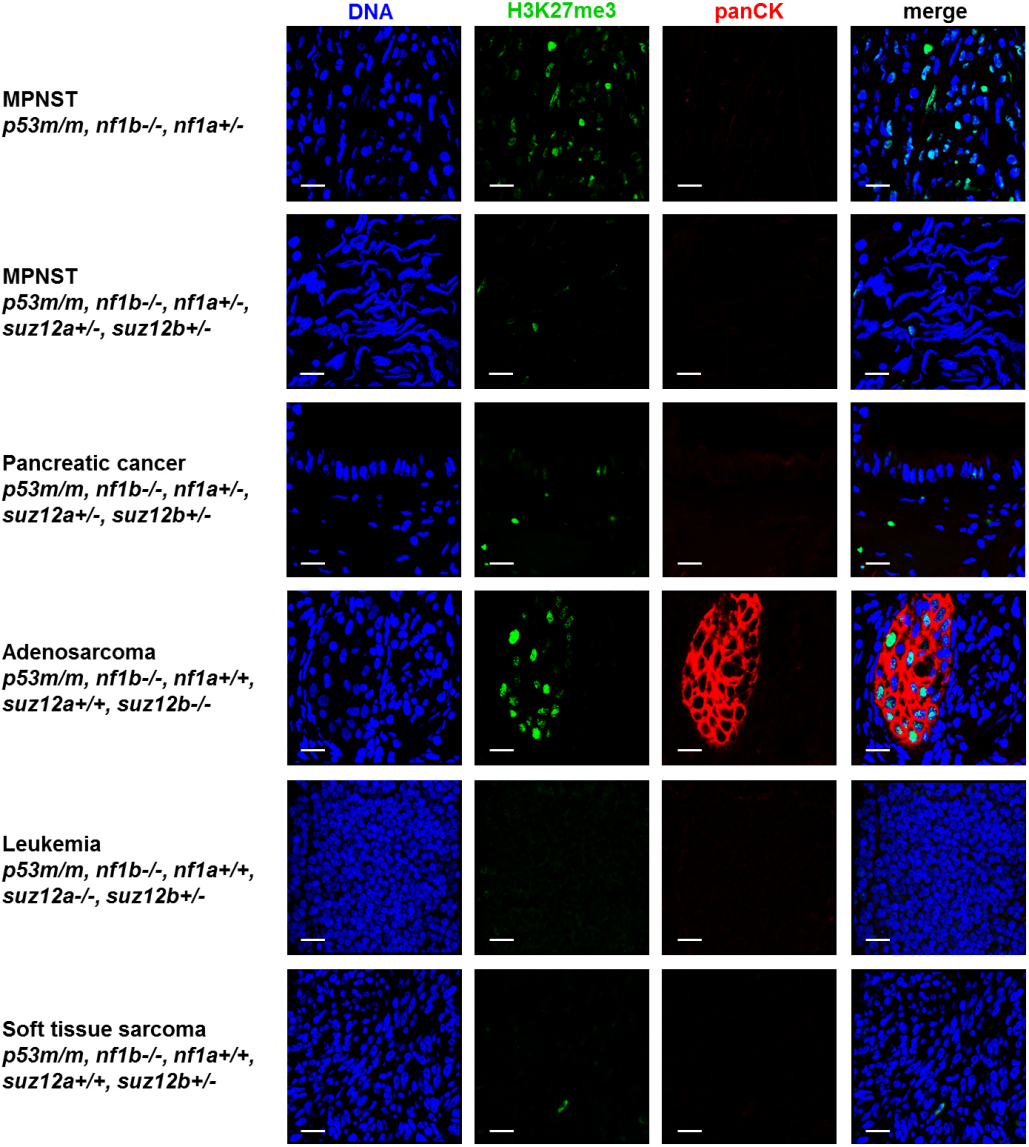
**Analysis of H3K27me3 and pan-cytokeratin by indirect immunofluorescence and confocal microscopy.** Paraffin-embedded sections of the indicated tumor types from zebrafish with the genotypes indicated were stained for H3K27me3 (green) and pan-cytokeratin (panCK; red). DNA is visualized using Hoechst 33342 (blue). Scale bars: 10 µm.

Because *suz12* encodes a core subunit of PRC2 that maintains silencing of target genes through the deposition of H3K27me3 marks, we examined the H3K27me3 status of *suz12*-deficient tumors. By staining tumor sections using indirect immunofluorescence, we observed a detectable H3K27me3 signal in *p53/nf1/suz12*-deficient MPNSTs, which was reduced compared to *p53/nf1*-defcient MPNSTs with functional *suz12* (Fig. 4, rows 1 and 2). The pancreatic adenocarcinoma, soft tissue sarcoma and leukemia cells each contained largely unstained nuclei (Fig. 4, rows 3, 5 and 6). By contrast, the mixed epithelial/mesenchymal adenosarcoma case displayed a heterogeneous H3K27me3 status with a strong signal in the epithelial glandular cytokeratin-positive compartment and a lack of H3K27me3 in the mesenchymal spindle-like cells (Fig. 4, row 4).

**Fig. 5.**
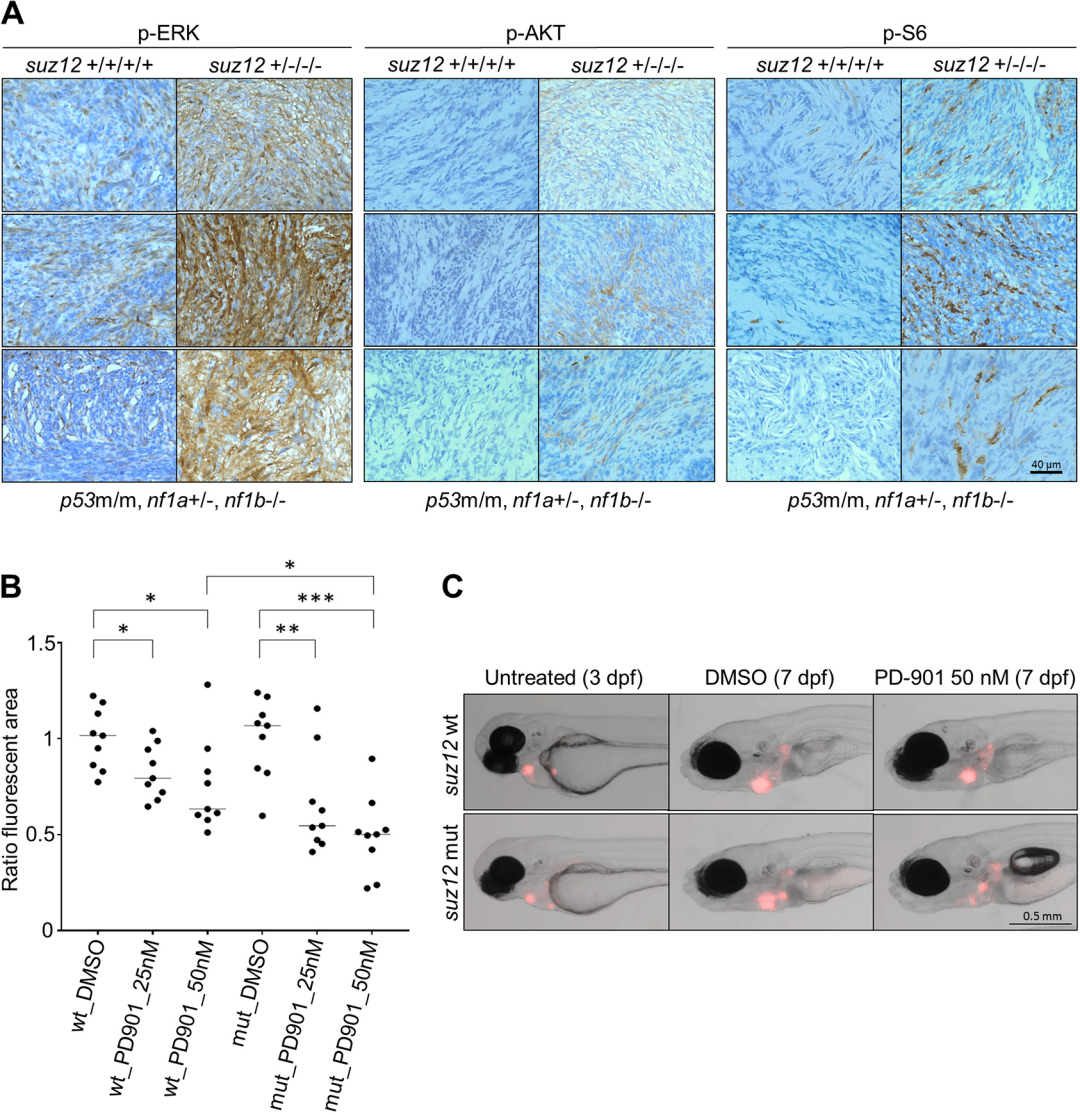
**Evaluation and inhibition of the Ras signaling pathway in *suz12*-mutant and *suz12*-wild-type MPNSTs.** (A) Immunohistochemistry analysis of signaling in three individual *suz12*-mutant (*suz12*^+/−/−/−^) and *suz12*-wild-type (*suz12*^+/+/+/+^) MPNSTs (*n*=3) each stained for phosphorylation of ERK, AKT, and S6 (p-ERK, p-AKT, and p-S6), indicating activation of Ras signaling. (B) The *suz12a^+/−^, suz12b^−/−^* (mut) or *suz12-*wild-type control MPNST (wt) tumor cell growth in the pericardial cavity of implanted embryos. These embryos were treated with DMSO vehicle control or PD-3025901 (PD901; 25 or 50 nM) (*n*=9 fish per treatment, doses based on the maximum tolerated dose of the individual drug). The fluorescent tumor area was determined for each embryo at 3 dpf (pre-treatment) and 7 dpf (post-treatment), and was reported as the normalized ratio of the red fluorescent area at 3  dpf versus 7 dpf in individual embryos. Individual values with medians (black bars) are shown. *P<0.05, **P=0.0064, ***P<0.0001 (Student's *t*-test). (C) Representative fish images at 3 dpf and 7 dpf after DMSO control or 50 nM PD0325901 treatment.

The reduced H3K27me3 modification upon *suz12*-depletion would be expected to affect gene expression on a global scale. Thus, we performed RNA-seq on *p53^m/m^, nf1b^−/−^, nf1a^+/^^−^, suz12-*mutant MPNSTs (*n*=4) and *p53^m/m^, nf1b^−/−^, nf1a^+/^^−^, suz12-*wild-type control MPNSTs (*n*=3). The control samples were derived as part of a previous study (Oppel et al., 2019). The results demonstrated elevated expression of gene sets representing PRC2 targets and gene sets related to oncogenic Ras signaling in *suz12*-deficient MPNSTs compared to *suz12*-wild-type MPNSTs in the *p53^m^*^/*m*^*,* *nf1b*^−/−^*,* *nf1a*^+/−^ background (Table 2, Tables S4 and S5). Similar results were obtained when we compared the gene expression profile of *p53/nf1/suz12*-deficient and *p53/nf1/atrx*-deficient tumor samples derived from a previous study (Table 2, Tables S6 and S7). To assess Ras-Mapk pathway signaling in *suz12* mutant MPNSTs compared to wild-type MPNSTs, we performed immunohistochemistry staining to qualitatively detect phosphorylation levels of Erk, S6 (Rps6) and Akt (p-Erk, p-S6 and p-Akt). In this analysis, *suz12*-deficient MPNSTs showed much stronger phosphorylation levels of all three factors (Fig. 5A), indicating increased Ras-Mapk signaling. Thus, in our *suz12*-knockout zebrafish model, impaired PRC2-mediated gene silencing cooperates with loss of *nf1* to increase signaling through the Ras-Mapk pathway.

**Table 2.**
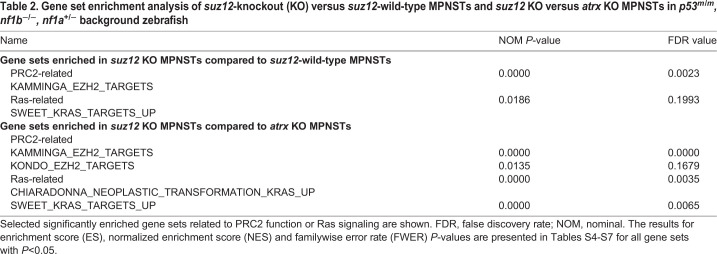
**Gene set enrichment analysis** **of *suz12*-knockout (KO) versus *suz12*-wild-type MPNSTs and *suz12* KO versus *atrx* KO MPNSTs in *p53^m^*^/*m*^*,nf1b*^−/−^*,nf1a*^+/^^−^** **background zebrafish**

### Suz12-depletion sensitizes MPNST cells to pharmacological inhibition of MEK

Our results shown in Fig. 5A demonstrate increased Ras-Mapk signaling in MPNSTs arising in *suz12/nf1/p53* combined mutant fish compared to *nf1/p53* mutant fish harboring wild-type *suz12* genes. This indicated a potentially increased vulnerability of *p53^m/m^, nf1b^−/−^, nf1a^+/^^−^, suz12-*mutant tumors towards pharmacological inhibition of this pathway. To test this hypothesis, we employed a previously described *in vivo* transplantation assay in living zebrafish embryos (Ki et al., 2019). For this assay, single cells were isolated from two groups of matched MPNST tumors: (1) *p53^m/m^,* *nf1b^−/−^,* *nf1a^+/−^, suz12*-mutant MPNSTs and (2) *p53^m/m^, nf1b^−/−^, nf1a^+/−^, suz12*-wild-type MPNSTs. These tumor cells were positive for a *sox10:mCherry* marker gene and injected into the pericardial cavity of transparent Casper recipient zebrafish embryos at 2 dpf. After 24 h, the injected cells had formed a fluorescent mass, and the embryos were treated with the MEK inhibitor PD-0325901 or with DMSO as a control. At 7 dpf, the tumor mass size was measured before and after treatment, and change in tumor area was compared in the MEK inhibitor-treated embryos and the DMSO-treated control embryos. We observed that MEK inhibition resulted in a decreased tumor size for both *p53^m/m^, nf1b^−/−^, nf1a^+/^^−^, suz12*-mutant MPNSTs and *p53^m/m^,* *nf1b^−/−^, nf1a^+/^^−^, suz12*-wild-type MPNSTs (Fig. 5B,C). However, when the responses of the MEK inhibitor-treated *suz12*-mutant tumors were compared to the *suz12*-wild-type tumors at 7 dpf, the *suz12*-mutant tumors were significantly smaller (Fig. 5B,C), indicating an increased dependency on high levels of Ras-Mapk pathway signaling in MPNSTs with loss of *suz12* function.

## DISCUSSION

### Knockout of *suz12* accelerates tumor development in cooperation with the *p53*/*nf1*-deficient background

Knockout of *suz12a* and *suz12b* in the zebrafish germline using CRISPR-Cas9 was highly efficient, and the vast majority of F1 fish examined carried a target-locus mutation in both genes, consisting mainly of deletions spanning less than 10 bp. Using this strategy, we were able to establish two *suz12a*- and *suz12b*-knockout lines using distinct sgRNAs that shared no sequence similarity. Both *suz12*-deficient lines demonstrated a strongly accelerated overall tumor onset and penetrance, in both the *p53^m/m^, nf1b^−/−^, nf1a^+/+^* and the *p53^m/m^, nf1b^−/−^, nf1a^+/^^−^* backgrounds. These results correspond well with observations in a previous mouse model of combined *Tp53/Nf1/Suz12* LOF (De Raedt et al., 2014). In that study, *Suz12* LOF was found to cooperate closely with *nf1* in MPNST development. We observed the same relationship in the present study, evident by significantly accelerated tumor onset in all *suz12-*depleted populations and 90-100% incidence of MPNSTs in tumor-bearing fish examined using histology.

Because both *nf1* and *suz12* are duplicated in zebrafish and there are four alleles each gene, we were able to determine the extent of cooperation between these genes at 25%, 50% and 75% doses. Based on previous studies, it is clear that *p53*-deficient zebrafish are already prone to delayed-onset MPNSTs and that the additional loss of *nf1* accelerates MPNST formation (Shin et al., 2012; Berghmans et al., 2005). However, the *p53^m/m^, nf1b^−/−^, nf1a^+/+^* genotype is only subtly more oncogenic than the *p53^m/m^* background. Only the loss of a third *nf1* allele (*nf1a*^+/−^) switches this line to a high-penetrance MPNST model (Shin et al., 2012). Interestingly, we found that a reduction in *suz12* gene dosage of only 25% is sufficient in zebrafish to cause a significantly accelerated onset and increased penetrance of tumors in the context of loss of both *nf1* and *p53*, regardless of which of the two *suz12* genes was inactivated on one allele (Fig. 2). Thus, mutating two or even three *suz12* alleles (Fig. 2) had rather little additional effect over mutating just one allele on the time of tumor onset or tumor penetrance. Apparently, the optimal concentration of Suz12 proteins in the cell is rate limiting, such that a threshold concentration expressed from all four alleles of *suz12* is critical for formation of the PRC2 complex, which contains Ezh2, Suz12, Eed and Rbap48. These tumor onset curves suggest that after one *suz12* allele is lost, there will be little selection pressure in somatic cells to drive the outgrowth of clones that have lost additional alleles through somatic mutation or silencing. Seemingly, loss of one allele representing a quarter of the normal gene dosage is sufficient to deplete the PRC2 complex, relax repression of self-renewal and proliferation genes, and thus promote the onset of tumors in the *nf1*/*p53*-depleted background.

Immunostaining of H3K27me3 in paraffin-embedded sections of tumors arising in these zebrafish supports this hypothesis (Fig. 4). The soft tissue sarcoma shown in Fig. 4 (row 6) developed in a fish with loss of only one allele of *suz12b*, and immunostaining shows only very minimal staining for H3K27me3. The H3K27me3 levels were also very low in an MPNST (Fig. 4, row 2) as well as a pancreatic cancer (Fig. 4, row 3) with loss of two *suz12* alleles, and a leukemia with loss of three *suz12* alleles (Fig. 4, row 5). Among the tumors in *suz12* mutant fish, only an adenosarcoma with loss of two alleles had high levels of staining for H3K27me3. Thus, there appears to be a tissue-specific influence, but in general loss of one allele of *suz12* can have profound effects on the levels of the repressive mark H3K27me3 in the nucleus, as detected by immunostaining. We do not have an antibody that recognizes zebrafish *suz12.* In the future, after a specific antibody is available, it will be possible to directly assess the levels of *suz12* expression in individual tumors and correlate these with levels of the H3K27me3 repressive mark.

Moreover, multiple tumor foci were observed only in zebrafish within the *suz12*-deficient cohort, and this result was only significant in *p53^m/m^, nf1b^−/−^, nf1a^+/^^−^, suz12-*mutant populations. In our experiments, this phenomenon was not detected in *p53/nf1*-deficient, *suz12*-wild-type control fish. The presence of multiple tumor foci can be attributed to either simultaneous onset of distinct tumors or metastatic dissemination. Loss of *SUZ12* has been linked directly to increased metastasis in gastric cancer and non-small cell lung cancer (Xia et al., 2015; Liu et al., 2014), suggesting that the multiple tumor foci with the same histology could in part be due to early dissemination from a single primary tumor.

### *suz12* LOF broadens the tumor spectrum in *p53/nf1*-deficient zebrafish

In the *p53^m/m^, nf1b^−/−^, nf1a^+/+^* or *nf1a*^−/+^ backgrounds, we observed a strong diversification of tumorigenesis upon loss of the important epigenetic regulator tumor suppressor *suz12.* By contrast, loss of the Ras-inactivating tumor suppressor *nf1 *in* suz12*-wild-type fish mainly accelerated the onset of MPNSTs, while inducing none of the other neoplasms observed in our study (Shin et al., 2012). Notably, a recently described zebrafish model based solely on the full deletion of *p53* (*p53^del/del^*) was prone to generate a broad spectrum of tumors, including leukemias (Ignatius et al., 2018).

Loss of SUZ12 promotes the onset of a variety of malignancies, including blood cancer subtypes (De Raedt et al., 2014; Ntziachristos et al., 2012; AACR Project GENIE Consortium, 2017). Most previously described specific models of leukemia in zebrafish have been driven by Rag2-mediated overexpression of *MYC*, *Akt2* and *NOTCH1* (Chen et al., 2007; Harrison et al., 2016; Gutierrez et al., 2011; Feng et al., 2010). Moreover, the leukemia penetrance of 20% provides a workable model of human leukemia with these mutations for future studies and the potential to specify leukemia subtypes.

### Partial *suz12* knockout decreases PRC2 activity and H3K27me3 deposition

All of the viable progeny of our breeding protocols still had at least one functional *suz12* allele, because a total knockout of each of the four genes was lethal in development. In the tumors, it is likely that additional *suz12* alleles are inactivated either by somatically acquired mutations, deletions or silencing. Thus, we think that the loss of H3K27me3 detected by immunofluorescence staining, as shown in Fig. 4, reflects the strong selection against these suppressive epigenetic marks in the multistep clonal selection that occurred during transformation of these primary tumors. These observations indicate that even subtle disturbances in the relative abundance of single PRC2 subunits can affect the ability of PRC2 to maintain the silencing of key target genes. This is supported by previous studies indicating that epigenetic regulator complexes such as PRC2 or SWI/SNF are sensitive to the stoichiometry of single subunits (Kadoch and Crabtree, 2015; Kloet et al., 2016). *NF1* functions as a suppressor of Ras signaling, whereas *SUZ12* is essential for genome-wide gene silencing of PRC2 targets and thus might have broad influence on multiple cellular processes including proliferation and differentiation (Margueron and Reinberg, 2011). This might explain why, in contrast to *nf1*, single allele loss of *suz12* in zebrafish is sufficient to promote the initiation of malignant tumors in a sensitized background.

As expected, zebrafish tumors with loss of *suz12* exhibited decreased H3K27me3 and upregulation of PRC2 target gene sets. As described previously, MPNSTs characterized by H3K27me3 loss have worse survival rates than tumors retaining this epigenetic mark (Cleven et al., 2016; Prieto-Granada et al., 2016). This observation correlates well with the faster onset and higher penetrance of MPNSTs developing in our model upon *suz12* inactivation. However, not all malignancies are promoted by a loss of PRC2 function. Certain types of breast cancer are known to harbor elevated PCR2 activity (Jang et al., 2016; Guo et al., 2016). Thus, our model appears to be consistent with a tumor spectrum that is driven by the upregulation of oncogene expression resulting from PRC2 loss, namely MPNSTs (De Raedt et al., 2014; Cleven et al., 2016) and leukemia (Ntziachristos et al., 2012). Our data further support the view that some types of leukemia are driven by the global loss of H3K27me3 (Ntziachristos et al., 2012). This mechanism extends to other low-penetrance cancer types observed in our model, such as pancreatic cancer. In human pancreatic cancer, lower H3K27me3 levels can predict a worse prognosis (Wei et al., 2008). In contrast, very little is known about the factors underlying the prognosis of soft tissue sarcomas. Our study suggests that loss of PRC2-mediated maintenance of gene expression might play a role in the multistep pathogenesis of human soft tissue sarcoma.

Interestingly, the adenosarcoma observed in our model displayed heterogeneous H3K27me3 staining, with intensely positive nuclei in the epithelial compartment and light to negative staining in the mesenchymal compartment. One hypothesis to explain this unusual finding in the epithelial compared to the mesenchymal components of this tumor is based on the presence of two functional alleles of *suz12* in the zebrafish. Because this fish has a germline genotype of *suz12a*^+/+^*,* *suz12b*^−/−^, one possibility is that the cytokeratin-positive epithelial cells express higher levels of *suz12a* in the nucleus and depend on *suz12a* for the formation of PRC2 complexes with Ezh2, Eed, and Rbap46. Thus, the epithelial component of the tumor contains abundant H3K27me3 histone modifications in the nucleus. According to this hypothesis, mesenchymal cells might naturally express much higher levels of *suz12b*, such that loss of *suz12b* in this fish would lead to the absence of detectable H3K27me3 histone modifications in these cells. Once specific antibodies for zebrafish Suz12a and Suz12b proteins are available, we will be able to address this hypothesis.

### Loss of *suz12* elevates Ras-Mapk signaling and sensitizes MPNSTs to MEK inhibition

Upon loss of combinations of one to three alleles of *suz12a* or *suz12b* in the zebrafish germline, we observed significant acceleration of MPNST onset and penetrance accompanied by the onset of additional tumor types. It is known from the murine system that diminished PRC2 function caused by *Suz12* knockout leads to elevated RAS signaling, which promotes MPNST development by amplifying RAS-driven transcription due to modulation of the chromatin structure (De Raedt et al., 2014). In the zebrafish system, our studies show that partial loss of *suz12* reduces silencing of PRC2 target genes and also activates the Ras-Mapk signaling cascade. It is reasonable to conclude that increased RAS-MAPK signaling initiated by the loss of PRC2-mediated transcriptional repression synergizes in tumorigenesis with loss of NF1, a potent deactivator of oncogenic RAS (Cawthon et al., 1990; Cichowski and Jacks, 2001; Viskochil et al., 1990; Wallace et al., 1990), because in this situation elevated RAS activation is combined with an impaired ability of cells to turn off RAS. This suggests that *NF1*-deficient MPNSTs carrying an additional loss of *SUZ12* are more dependent on oncogenic RAS-MAPK signaling than *SUZ12*-wild-type MPNSTs. Indeed, in zebrafish we observed an increased sensitivity to MEK-inhibition in *suz12*-mutant MPNSTs in the *p53/nf1*-deficient background, which is in accordance with previous observations on the effects of MEK inhibition in MPNSTs (Ki et al., 2019; Jessen et al., 2013), especially in cooperation with the BRD4-inhibitor JQ1 (De Raedt et al., 2014). It has previously been described in the murine system that *Suz12* acts as a tumor suppressor in *Nf1*-deficient but not in *Nf1*-wild-type tumors (De Raedt et al., 2014). In combination with our findings, this indicates that simultaneous loss-of-function mutations or deletions of the tumor suppressors *SUZ12* and *NF1* might be a marker for the clinical use of molecular targeted drugs against MPNSTs that inhibit the RAS-MAPK pathway, for example MEK inhibitors such as Trametinib, Cobimetinib, and Binimetinib. This could be investigated in future clinical trials.

In mice, it is known that combined deficiencies in *Tp53* and *Nf1* synergize in the onset of MPNSTs and high-grade gliomas, and that the combined loss of *Suz12* and *Nf1* cooperate in the initiation of MPNSTs without loss of *Tp53* (De Raedt et al., 2014). Our zebrafish *p53/nf1/suz12*-deficient model was created from the *p53/nf1*-knockout zebrafish line described previously (Shin et al., 2012). The mutational and deletional inactivation of all three of these genes occurs in at least 28% of human MPNST tumors (Lee et al., 2014), making this a very important genotype in MPNST biology. In future studies, it will also be important to determine whether *nf1/suz12* loss in *p53-*wild-type zebrafish will also promote the onset of MPNSTs, as is the case in mice (De Raedt et al., 2014).

The impact of loss of *p53* in this genetic context remains unclear. It is known that the combined deficiencies in *p53* and *nf1* synergize in the onset of MPNSTs and high-grade gliomas (Shin et al., 2012), and that the combined deficiencies in *Suz12* and *Nf1* cooperate in widespread tumor development in mice (De Raedt et al., 2014). Thus, we believe that the loss of p53 further promotes, but is not essential for, the synergistic effects of deficiencies in *nf1* and *suz12*. However, because our model does not allow the distinction between the *p53/nf1/suz12* mutant and the *nf1/suz12*-mutant background, this has to be explored in future studies.

In summary, we show that the role of PRC2 in tumor suppression is very sensitive to the dosage of *suz12* in multiple tissues and that complete loss of *suz12* is not required to promote tumorigenesis. The inactivation of one or more alleles of *suz12* in zebrafish with an *nf1*/*p53* sensitized genetic background accelerates tumor onset and expands the spectrum of tumors in a fashion consistent with genetic abnormalities found in human cancers. Thus, the consequences of loss of H3K27me3 marks maintained by PRC2 during oncogenesis might be conserved between zebrafish and humans, raising the possibility that important strategies to counteract these epigenetic alterations can be investigated in zebrafish models, ultimately leading to the identification of specific molecules that antagonize the cancer-promoting effects of PRC2 deficiency. However, zebrafish is unlikely to be a good model of some tumors commonly associated with *suz12* mutations, such as penile, endometrial, and bladder carcinomas.

## MATERIALS AND METHODS

### Zebrafish strains and maintenance

All zebrafish (*Danio rerio*) strains used were either AB (wild type) background or *p53/nf1b/nf1a*-deficient background (Shin et al., 2012). These fish carry a homozygous *p53-M214K* mutation (*p53^m/m^*), as previously described (Berghmans et al., 2005). All zebrafish experiments and housing were performed according to Dana-Farber Cancer Institute IACUC-approved protocol #02-107.

### CRISPR-Cas9 genome editing

Zebrafish strains with germline mutations in *suz12* were generated by the CRISPR-Cas genome editing system (Hwang et al., 2013), using pCS2-nCas9n to transcribe *Cas9 in vitro*. The plasmid constructs pDR274 (Addgene #42250) and pCS2-nCas9n (Addgene #47929) were purchased from Addgene. The following sgRNA sequences were employed to target exon 1 of *suz12a* or *suz12b*: *suz12a*-sgRNA 1, 5′-GGAGGAGCTCACGCATCGTC-3′; *suz12a*-sgRNA 2, 5′-AGCCGACCACCAACTCTTCC-3′; *suz12b*-sgRNA 1, 5′-GTGAGCTCACGCCAGAAGAT-3′; *suz12b*-sgRNA 2, 5′-GGTGCTGTATACCCATCTTC-3′. All oligonucleotides were purchased from Eurofins Genomics (Louisville, KY, USA). To establish *suz12*-knockout line 1, we used sgRNAs 1 targeting exon 1 for *suz12a* and *suz12b* in combination (pair 11×11), and for *suz12*-knockout line 2 we used sgRNAs 2 targeting exon 1 for *suz12a* and *suz12b* in combination (pair 12×12).

### Injection in zebrafish embryos and genotyping

pDR274-sgRNA plasmid DNA was linearized with DraI (NEB, Ipswich, MA, USA), while sgRNA sequences were transcribed *in vitro* using the MAXIscript T7 Kit (Ambion Inc., Foster City, CA, USA). *Cas9* mRNA was transcribed *in vitro* from *pCS2-nCas9n* plasmid DNA linearized with NotI (NEB), using the mMessage mMachine SP6 Kit (Ambion Inc.). Oligonucleotides were mixed in ratios between 1:5 and 1:1 with a 0.5% Phenol Red solution (Sigma-Aldrich, Burlington, MA, USA) and set to a final concentration of 25 ng/µl sgRNA and 600 ng/µl *Cas9* mRNA. To induce mutations in the genome of zebrafish, we injected one-cell-stage embryos with the oligonucleotide/Phenol Red mix described above within 30 min after fertilization using a glass capillary mounted into an air pressure injector (Harvard Apparatus, Cambridge, MA, USA). The injection volume was 1 nl oligonucleotide/Phenol Red mix per one-cell-stage embryo. Dead embryos were removed at 3 to 6 h after injection.

Injected fish were raised as mosaics and crossed to identify germline mutations inherited into the F1 generation. Next, fish mutants (*suz12a* and *suz12b*) were bred together to establish stable *suz12*-deficient lines. To genotype the *suz12a*/*suz12b* mutant line, genomic DNA was isolated with QuickExtract DNA Extraction Solution (Epicenter; Madison, WI, USA). PCR was performed using Taq-polymerase (NEB) according to the standard protocol, with T_m_ of 60°C, and 40 cycles. Oligonucleotides used were: *suz12a* forward primer, 5′-AAACGTCTCGTTCGACCCC-3′; *suz12a* reverse primer, 5′-AGCCTTCAAGCGAGGAGTG-3′; *suz12b* forward primer, 5′-CGAGGGCGACTGTCTGTCAT-3′; *suz12a* reverse primer, 5′-CAACAGCACGTTGTCATGAACT-3′. The DNA products were sequenced with the forward or reverse primer.

### Tumor watch experiments

The *sox10:GFP* zebrafish were genotyped for mutations in *nf1a*, *suz12a* and/or *suz12b* at age 2-3 months and sorted into separate tanks by genotype. Alleles of *p53* and *nf1b* were maintained as homozygous knockouts. The fish were inspected every 1-2 weeks for visible tumors affecting any part of their bodies and also for abnormal behavior such as inactivity, abnormal or uncoordinated swimming, hovering near the bottom of the tank, or lack of aggressive feeding behavior at the daily feeding time. The fish in each tank were also counted to ensure fish were not unknowingly lost from the tanks. During the biweekly anesthetized examination of each fish under a Nikon C-DSD115 fluorescence microscope, the fish were also inspected using brightfield microscopy for evidence of a tumor mass or a pallor reflecting anemia. If abnormalities were detected, the time of onset was established and the fish were observed for two more weeks to make sure the findings persisted. Then, the fish were humanely euthanized on ice using tricaine, fixed in formalin and analyzed by histology for tumors after sectioning the entire fish. Tumor onset is defined as the first visual recognition of tumor growth not regressing within a time frame of 2 weeks. GraphPad Prism 7 software was used to conduct survival analysis and to calculate *P*-values by *t*-test. Photoshapes of tumor-bearing fish were taken using an iPhone 6. All fish that died before tumor onset were removed from the analysis.

### Histopathology analysis of zebrafish tissue

Tumor-bearing fish were sacrificed and subsequently fixed for 1-3 days in 4% paraformaldehyde diluted in PBS at 4°C. After fixation, fish were washed in PBS and stored in 70% ethanol until embedded in paraffin. Paraffin sectioning (3 μm) and Hematoxylin/Eosin (HE) staining was performed at the Dana-Farber/Harvard Cancer Center Research Pathology Core using standard protocols. Individual tumor-bearing fish examined by histopathology were randomly selected. Immunohistochemistry staining was performed as described previously (He et al., 2016) using the following primary antibodies: phospho-p44/42 MAPK (ERK1/2) (Thr202/Tyr204; Cell Signaling Technology #4370; 1:150), phospho-AKT (Ser473; Cell Signaling Technology #4060; 1:100), phospho-S6 ribosomal protein (Ser240/244; Cell Signaling Technology #4838; 1:100). Between 24% to 31% of all tumor-bearing fish from the *p53/nf1/suz12*-deficient population and 50% to 57% of the *p53/nf1*-depleted, *suz12*-wild-type control fish were analyzed.

### Indirect immunofluorescence staining

Indirect immunofluorescence staining was performed as described in previous studies (Oppel et al., 2019, 2011; Ball et al., 2017). Primary antibodies used were pan-cytokeratin (AE1/AE3; Novus Biologicals #NBP2-29429; 1:200) and tri-methyl-Histone H3 (Lys27) (C36B11; Cell Signaling Technology #9733; 1:400). Secondary antibodies used were goat anti-rabbit-IgG and goat anti-mouse-IgG conjugated with Alexa 488 or 568 (Life Technologies; 1:400).

### RNA-seq analysis

RNA was isolated from half of the *p53/nf1/suz12*-deficient tumors, while the other half was analyzed by histopathology as described above. Four *p53^m/m^, nf1b^−/−^, nf1a^+/^^−^, suz12-*mutant (two *p53^m/m^, nf1b^−/−^, nf1a^+/^^−^, suz12a*^+/−^*, suz12b^+/−^* and two *p53^m/m^, nf1b^−/−^, nf1a^+/^^−^, suz12a*^+/−^*,* *suz12b^−/−^*) were analysed, as previously described (Oppel et al., 2019). As control samples, the RNA-seq data of three *p53^m/m^, nf1b^−/−^, nf1a^+/−^, suz12-*wild-type tumors of a previous study were used (Oppel et al., 2019). Quality control, library preparation, and next-generation sequencing was performed by the Molecular Biology Core Facility of the Dana-Farber Cancer Institute according to standard protocols. Bioinformatics gene set enrichment analysis was performed as previously described (Oppel et al., 2019). Gene sets related to PRC2 and RAS signaling were extracted manually from the analysis. RNA-seq data from the *p53/nf1*-deficient and the *p53/nf1/atrx*-deficient fish tumors were derived from a previous study (Oppel et al., 2019) and compared to data from the *p53/nf1/suz12*-deficient tumors from this study. RNA-seq data from Oppel et al. (2019) and from this study are available under Gene Expression Omnibus (GEO) accession number GSE125040 (updated 12 August 2020).

### AACR Genie database analysis

Data were extracted from the AACR Genie database (v4.0), September 2018, and further processed with Microsoft Excel.

## Supplementary Material

Supplementary information
