# Supplementary material for: suz12 inactivation in p53- and nf1-deficient zebrafish accelerates the onset of malignant peripheral nerve sheath tumors and expands the spectrum of tumor types
Source: Dis Model Mech. 2020 Aug 27;13(8):dmm042341. doi: 10.1242/dmm.042341 (PMC7473648; doi:10.1242/dmm.042341)
Supplement: Supplementary information [file dmm-13-042341-s1.pdf]

Table S1: Mutant genomic sequences of *suz12* target loci in F1 zebrafish

Click here to Download Table S1

Table S2: Histopathology analysis of tumor-bearing fish

Click here to Download Table S2

Table S3: Frequency of *SUZ12* mutations and copy-number alterations in human tumor samples annotated in the AACR Genie database (v4.0)

Click here to Download Table S3

Table S4: Gene sets significantly enriched in *p53/nf1/suz12*-deficient MPNSTs (p < 0.05, FDR < 0.25)

Click here to Download Table S4

Table S5: Gene sets significantly enriched in *p53/nf1*-deficient, *suz12*-wildtype control MPNSTs (p < 0.05, FDR < 0.25)

Click here to Download Table S5

Table S6: Gene sets significantly enriched in *p53/nf1/suz12*-deficient, *atrx*-wildtype MPNSTs (p < 0.05, FDR < 0.25)

Click here to Download Table S6

Table S7: Gene sets significantly enriched in *p53/nf1/atrx*-deficient, *suz12*-wildtype MPNSTs (p < 0.05, FDR < 0.25)

Click here to Download Table S7
